# Genome-wide methylation profiling of Peripheral T-cell lymphomas identifies TRIP13 as a critical driver of tumor proliferation and survival

**DOI:** 10.21203/rs.3.rs-3971059/v1

**Published:** 2024-02-29

**Authors:** Pawel Nowialis, Julian Tobon, Katarina Lopusna, Jana Opavska, Arshee Badar, Duo Chen, Reem Abdelghany, Gene Pozas, Jacob Fingeret, Emma Noel, Alberto Riva, Hiroshi Fujiwara, Rene Opavsky

**Affiliations:** University of Florida College of Medicine; University of Florida College of Medicine; University of Florida College of Medicine; University of Florida College of Medicine; University of Florida College of Medicine; University of Florida College of Medicine; UF College of Liberal Arts and Sciences, University of Florida; UF College of Liberal Arts and Sciences, University of Florida; UF College of Liberal Arts and Sciences, University of Florida; University of Florida; University of Florida; Ehime University Graduate School of Medicine; University of Florida College of Medicine

**Keywords:** cancer biology, DNA methylation, DNA methyltransferase, gene expression, lymphoma

## Abstract

Cytosine methylation of genomic DNA contributes to the regulation of gene expression and is involved in normal development including hematopoiesis in mammals. It is catalyzed by the family of DNA methyltransferases (DNMTs) that include DNMT1, DNMT3A, and DNMT3B.

Peripheral T-cell lymphomas (PTCLs) represent a diverse group of aggressive mature T-cell malignancies accounting for approximately 10–15% of non-Hodgkin lymphoma cases in the US. PTCLs exhibit a broad spectrum of clinical, histological, and immunophenotypic features with poor prognosis and inadequately understood molecular pathobiology.

To better understand the molecular landscape and identify candidate genes involved in disease maintenance, we used high-resolution Whole Genome Bisulfite Sequencing (WGBS) and RNA-seq to profile DNA methylation and gene expression of PTCLs and normal T-cells. We found that the methylation patterns in PTCLs are deregulated and heterogeneous but share 767 hypo- and 567 hypermethylated differentially methylated regions (DMRs) along with 231 genes up- and 91 genes downregulated in all samples suggesting a potential association with tumor development. We further identified 39 hypomethylated promoters associated with increased gene expression in the majority of PTCLs. This putative oncogenic signature included the *TRIP13* (thyroid hormone receptor interactor 13) gene whose both genetic and pharmacologic inactivation, inhibited cellular growth of PTCL cell lines by inducing G2-M arrest accompanied by apoptosis suggesting that such an approach might be beneficial in human lymphoma treatment. Altogether we show that human PTCLs are characterized by a large number of recurrent methylation alterations, and demonstrated that TRIP13 is critical for PTCL maintenance *in vitro*.

## Introduction

Non-Hodgkin’s lymphomas (NHLs) are a heterogeneous group of lymphoid malignancies that arise from the transformation of B-, T-, and NK cells. Although the majority of NHLs are B-cell lymphomas (BCLs), ~ 15% of patients in Western countries suffer from more aggressive Peripheral T-cell lymphomas (PTCLs) with poor survival rates^1–3^. PTCLs exhibit a broad spectrum of clinical, histological, and immunophenotypic features, which present significant therapeutic challenges and an overall unfavorable prognosis. Based on their manifestations - disseminated, nodal disease, extranodal, or cutaneous - and molecular features, at least 29 discrete types of PTCLs have been identified to date^4^.

The three most common PTCL subtypes are Anaplastic large cell lymphomas (ALCL), Angioimmunoblastic T-cell lymphoma (AITL), and PTCL-not otherwise specified (PTCL-NOS).

ALCL are CD30-positive aggressive lymphoproliferative disorders affecting lymph nodes and extranodal sites and characterized by the expression of the Anaplastic lymphoma kinase (ALK) protein as either ALK-positive and ALK-negative subtypes^5^. AITLs arise from the follicular T helper cells and manifest by liver and spleen enlargement, lymphadenopathy, and weight loss^4–5^.

Approximately 30% of PTCLs are “not otherwise specified” (PTCL-NOS) constituting the most common subtype. This group of high-grade mature T-cell neoplasms is not classified by other WHO criteria due to our poor understanding of their molecular makeup^4^. Patients with PTCL-NOS are diagnosed with disease confined to the lymph nodes with frequent involvement of the liver, spleen, bone marrow, gastrointestinal tract, and skin. PTCL-NOS cells have an aberrant expression of T-cell markers (e.g. the lack of CD7 expression) with either CD4+/CD8- or CD4-/CD8 + phenotype and have high proliferation rate^4,5^. Recently, two new subgroups of PTCL-NOS were identified: 1. *TBX21* expressing (PTCL-TBX21; 49% of cases) 2. *GATA3* expressing (PTCL-GATA3; 33% cases)^6^.

Due to the poor understanding of the disease, Peripheral T-cell lymphomas (PTCLs) are clinically treated with combination chemotherapy such as CHOP or CHOEP but with limited success^4,5^. For example, PTCLs, have a low 32% 5-year survival rate^3^.

Cytosine methylation in mammalian genomic DNA is an epigenetic mark that along with covalent histone modifications contributes to the suppression of gene promoter activity and repetitive elements resulting in transcriptional repression^7,8^. It can also lead to more efficient binding of transcription factors to their recognition sites as well as restricting the activation of alternative promoters within gene bodies thereby promoting transcription^9,10^. The family DNA methyltransferases (DNMT1, DNMT3A, DNMT3B, DNMT3L; DNMTs) are primarily responsible for catalyzing an addition of methyl group to cytosines in CpG dinucleotides in DNA on a genome-wide level. DNMT1 is considered to be the main maintenance enzyme while DNMT3A and DNMT3B are primarily involved in *de novo* activities^11–13^. DNMT3L lacks catalytic activity but serves as a critical accessory protein for de novo methylation^14^. Several other proteins are important in the regulation of cytosine methylation. The TET enzymes are a family of methylcytosine dioxygenases and are critical for DNA demethylation. Another protein that can affect DNA methylation is TCL1A protein which inhibits DNMTs biochemically and affects global methylation in a mouse model of hematological malignancies^15^.

Given the importance of methylation modifiers in regulation of transcription it is not surprising that many players are mutated in hematologic malignancies. Most point mutations in DNMT3A and TET2 are frequently found in hematologic malignancies including PTCL and result in loss of functions^16^. Consistently, loss of function mouse models demonstrated that Dnmt3a, Dnmt3b, and Tet2 are tumor suppressors in a variety of hematologic malignancies including T- and B-cell lymphomas^17–23^. The molecular landscape of mouse lymphomas was characterized by global hypomethylation affecting all parts of the genomes including repeat elements, promoters, enhancers, gene bodies, and intergenic regions and contributing to de-repression. In contrast, locus-specific hypermethylation in promoters resulted in gene silencing of a large number of genes^17–23^.

Comprehensive DNA methylation profiling can provide a wealth of information on molecular changes altered in tumors which, in turn, can be used for the development of diagnostic and prognostic markers, and lead to the identification of key drivers involved in the initiation and progression of PTCL. Given the value of such information, cancer-specific DNA methylation patterns of several human PTCL types have been partially characterized to date. In subtypes of human ALCL, DNA methylation signatures affect TCR and other genes involved in T-cell differentiation, as well as transcription factor induction and occupancy thereby contributing to ALCL oncogenic signaling^24^. In hepatosplenic TCL, several hyper- or hypomethylated loci were identified in gene regulatory elements including enhancers with eight-gene signatures of recurrent changes^25^.

To better understand the recurrent molecular changes, and their possible contribution to disease maintenance, here we used high-resolution methylation and gene expression profiling of up to 10 PTCLs along with normal T-cells.

We found that whereas normal T-cell methylation patterns are quite similar across several samples, methylation patterns in PTCL are heterogeneous and characterized by both hypo- and hypermethylated regions relative to normal controls with the majority of changes not shared across tumors. In addition, we identified methylation (767 hypo - and 567 hypermethylated DMRs) and gene expression signatures (31 genes up- and 91 genes down) present in all tested tumors suggesting a potential association with tumor development. We also found that putative tumor suppressors, such as *FOXP1, STK4*, and *ATM were* frequently hypermethylated and silenced whereas oncogenes, such as *UHRF1, MPZL1, CDK14, RACGAP1*, and *RAB13*, were hypomethylated and overexpressed suggesting that DNA methylation changes contribute to PTCL development. Among putative oncogenes was alsoTRIP13 (thyroid hormone receptor interactor 13) hypomethylated and overexpressed in 9/10 PTCLs on average of ~ 10- fold. We show that both TRIP13 knockdown and its pharmacological inhibition by a small-molecule inhibitor DCZ0415 impaired cellular growth of T-cell lymphoma cell line T8ML-1^26^, as well as T-cell leukemia cell lines by inducing G2-M arrest and apoptosis suggesting the role of this gene in maintenance of T-cell malignancies.

Altogether we show that human PTCL are characterized by a large number of recurrent methylation alterations affecting the expression of genes involved in tumorigenesis and functionally demonstrate that TRIP13 plays a role in PTCL lymphoma maintenance *in vitro*. Therefore, targeting TRIP13 might be beneficial in human PTCL treatment.

## Results

### DNA methylome and transcriptome of normal human CD4 + and CD8 + T-cells

**Figure 1:**
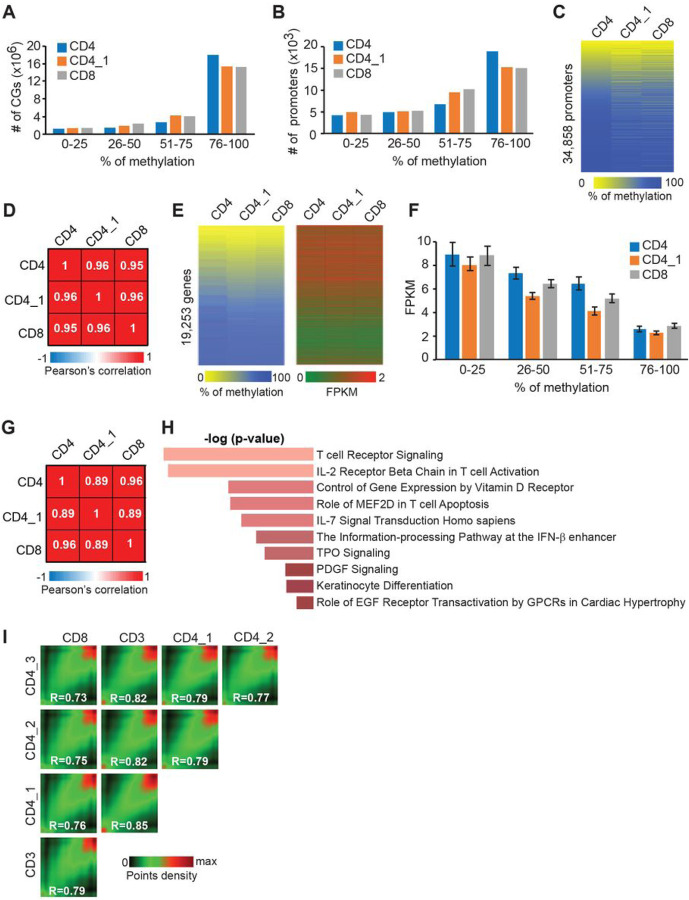
DNA methylome and transcriptome of normal human T-cells **(A)** Breakdown of CpG methylation as determined by WGBS in two independent CD4+ (CD4, CD4_1) and one CD8+ (CD8) normal human T-cell samples. Individual CpGs were placed into four categories based on percent methylation (0–25%, 26–50%, 51–75%, and 76–100%). **(B)** Breakdown of promoter methylation for 34,858 genes in CD4+ and CD8+ normal human T-cell samples. Methylation percentages for all CpGs across the 2,000 bp region (−1,500 bp to +500 bp relative to TSS) were averaged to give a mean methylation value for each gene promoter. Promoters were placed into four categories based on percent methylation (0–25%, 26–50%, 51–75%, and 76–100%). **(C)** Methylation status of 34,858 promoters in CD4+ and CD8+ samples determined by WGBS. Mean promoter methylation was determined as described in **B**. A scale is shown below the heat map, in which yellow and blue correspond to a lower and higher methylation status, respectively. **(D)** Pearson correlation coefficients (R values) derived from pairwise comparisons of 34,858 promoter methylation between CD4+, CD4_1, and CD8+ samples. Values > 0.9 indicate a strong positive correlation. **(E)** Heat map displaying methylation status of 19,253 promoters in normal human CD4+ and CD8+ cells as determined by WGBS (analyzed as in **B)** and corresponding gene expression (FPKM values obtained by RNA-seq). A scale is shown below the heat map, in which yellow and blue correspond to a lower and higher methylation status, respectively. The corresponding expression is shown as a heat map with highly expressed genes denoted in red and lowly expressed genes denoted in green. **(F)** Analysis of promoter methylation and gene expression in normal human T-cells for 19,253 genes. Genes were divided into four groups based on the percentage of promoter methylation (0%−25%, 26%−50%, 51%−75%, and 76%−100%). Bars represent Mean +/−SEM FPKM. Pairwise comparisons were made for four methylation groups within each sample. All differences were statistically significant (p < 0.05, two-tailed Student’s t-test) except 0%−25% vs 26%−50% and 26%−50% vs 51 %−75% in the CD4+ (blue) sample. **(G)** Pearson correlation coefficients (R values) derived from pairwise comparisons of gene expression values of 19,253 genes in normal CD4+ and CD8+ T-cells based on RNA-seq data. Values > 0.85 indicate a strong positive correlation. **(H)** Pathway enrichment analysis of 3,001 highly expressed genes (FPKM ≥ 5) by *EnrichR*. The most significant pathways in *“Biocarta 2016”* are shown (p < 0.05). **(I)** Pairwise comparison of CpG methylation in normal CD4+ (n = 3), CD8+ (n = 1), and CD3+ (n = 1) T-cells. The density of points increases from green to red. R values represent Pearson’s correlation coefficients.

To identify molecular changes associated with PTCL development, we first sought to determine methylation and gene expression patterns in normal T-cells. To achieve that we obtained CD4 + and CD8 + normal human T-cell samples isolated from peripheral blood (Precision Medicine) and subjected them to Whole Genome Bisulfite Sequencing (WGBS) and RNA-seq analysis. WGBS yielded more than 28M reads for each sample (**Fig. S1**). We next analyzed methylation data along with data obtained from purified CD4 + T-cells (sample CD4_1) for which both WGBS and RNA-seq were publicly available. The methylation analysis revealed that 21,936,712 individual CpG dinucleotides (CGs) were covered ≥ 5x in all three samples and were used further. A majority of CGs had a high level of methylation with more than 1.4 × 10^7^ CGs methylated to at least 75% in all three samples, whereas only 1 × 10^6^ CGs were methylated ≤ 25% (Fig. 1A). Further analysis revealed that more than 15,000 out of 34,858 promoters (defined as ‒ 1500 bp to + 500 bp relative to the transcription start site -TSS) were heavily methylated (at least 75%) and only ~ 4,000 promoters were methylated at lower levels (less than 25%) in each sample (Figs. 1B, 1C **and Supporting Information 1**). The positional variability in promoter methylation was low with very similar patterns across samples as determined by Pearson’s correlation with R values close to 1 in all pairwise comparisons (Fig. 1D). We next utilized RNA-seq data to determine if methylation may impact gene expression. This analysis revealed that in general, the degree of promoter methylation is inversely correlated with gene expression (Figs. 1E, 1F **and Supporting information 2**). Pairwise comparisons of gene expression and methylation revealed that FPKM values for genes with different percentages of promoter methylation were significantly different (p < 0.05, two-tailed Student’s t-test) in all comparisons except 0–25% vs 26–50% and 26–50% vs 51–75% in the first CD4 + T-cells sample (Fig. 1F). For example, genes with promoters that were less than 25% methylated were expressed at higher levels than genes with highly methylated promoters (>75%; Fig. 1F). Three analyzed T-cell samples were more variable in gene expression patterns as R values in pairwise comparisons were lower than those seen based on promoter methylation (Fig. 1G). This result suggests that promoter methylome is more stable than transcriptome in normal T-cells across individuals and perhaps less prone to polymorphic changes in the human population.

Pathway enrichment analysis of 3,001 highly expressed genes (FPKM ≥ 5) by *EnrichR* revealed, not surprisingly, significant enrichment in genes related to T-cell development. The most significant in *Biocarta 2016 were* T-cell receptor, IL2 -, IL7- signaling, and T-cell apoptosis (Fig. 1H).

To further investigate methylation variability in human T-cells, we analyzed the similarity of methylation patterns among an additional three samples for which WGBS data are available. A pairwise comparison of the methylation status of 7,628,019 cytosines (covered 5x) that overlapped in all controls revealed that samples showed a high degree of similarity irrespective of their immunophenotypes (R = 0.73–0.85) for all pairwise comparisons (Fig. 1I).

Our analysis thus demonstrates that a majority of CpG dinucleotides and promoters are mostly methylated in normal T-cells and that methylation polymorphism is limited resulting in relatively infrequent differences in promoter methylation between CD4 + and CD8 +T-cells.

### DNA methylome of Peripheral T-cell lymphomas

To analyze DNA methylation in human PTCL, we first obtained a set of seven primary human lymphoma samples from both the Cooperative Human Tissue Network (CHTN) and commercial sources. This resulted in a collection of four ALCL (T1-T4), one AITL sample (T5), and two PTCL-NOS (T6, T8) (**Fig. S2**). After DNA isolation, we subjected the obtained samples to global methylation profiling using WGBS.

**Figure 2:**
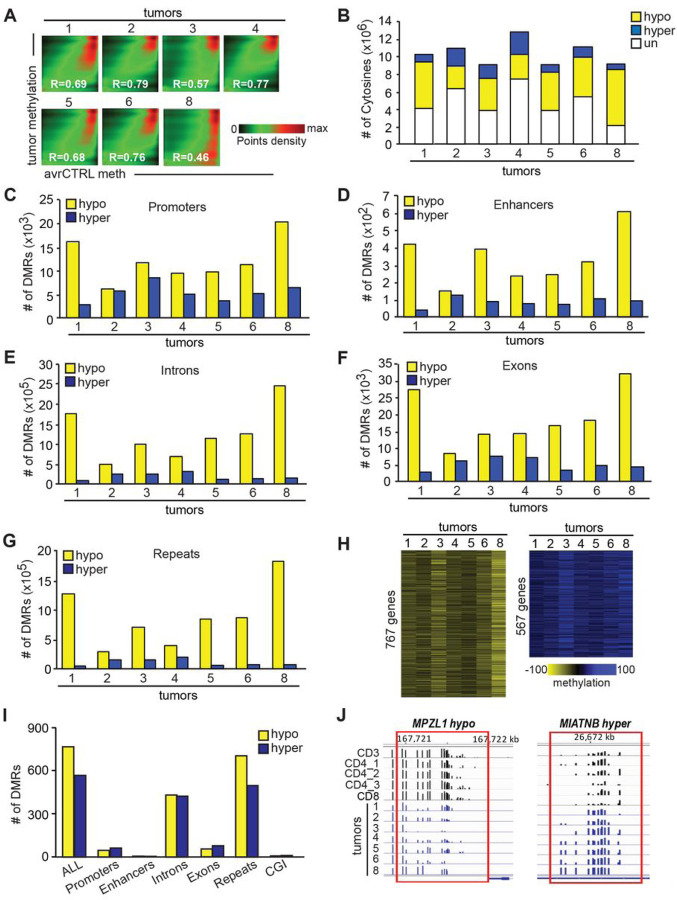
DNA methylome of human peripheral T-cell lymphomas **(A)** Pairwise comparison of CpG methylation in human PTCLs against the average of five normal T-cell controls (CD3, CD4_1, CD4_2, CD4_3, CD8) averaged out by *Metilene*. The density of points increases from green to red. R values represent Pearson’s correlation coefficients. **(B)** The number of hypomethylated (yellow), hypermethylated (blue), and unchanged (white) CpGs in human PTCLs when compared to respective normal controls. (meth. diff. ≥ 10%). **(C)** The numbers of hypomethylated and hypermethylated DMRs in promoter regions of human PTCLs when compared to normal controls (DMRs; regions with methylation difference ≥ 10% in the same direction in ≥ 3 consecutive DMCs separated by < 100 bp; p (MWU) < 0.05). The numbers of hypomethylated and hypermethylated DMRs in enhancers **(D)**, introns **(E)**, exons **(F)**, and repetitive elements **(G)** of human PTCLs when compared to normal controls. **(H)** Heat maps of the ‘Core PTCL Methylation Signature’ containing 767 hypo- and 567 hypermethylated DMRs present in all seven PTCLs tested. DMRs were analyzed as described in the **C** part of this figure. A scale of *%* methylation relative to the average of controls is shown below the heat map. Yellow and blue correspond to a lower and higher methylation status, respectively. **(I)** Distribution of DMRs in the ‘Core PTCL Methylation Signature’ across indicated genomic elements. **(J)** Percentage of methylation at individual CpGs in DMRs at *MPZL1* locus *(left)* and *MIATNB* (right) at indicated genomic coordinates consistently hypo- and hypermethylated in PTCLs, respectively, as visualized by IGB software.

Analysis of the data revealed that we were able to obtain sequencing depths ranging from 16.5 to 24M CGs that were covered by at least five sequence reads in each sample (**Fig. S3**). For analysis of tumor-specific methylation, we used control sample consisting of methylation data obtained from five normal human T cells - CD3, CD4_1, CD4_2, CD4_3 and CD8 - averaged out by *Metilene*. A pairwise comparison of the methylation status of 7,628,019 cytosines (covered 5x, averaged out by *Metilene)* that overlapped in all samples revealed that tumors were severely hypomethylated relative to the controls (Figs. 2A, S1 and S3). This was further confirmed by the analysis of differentially methylated cytosines (DMCs) which were those that had at least 10% methylation change relative to controls (p < 0.05 (MWU)). Our pairwise analysis on CGs covered in all samples revealed that most of the DMCs were hypomethylated relative to normal T-cells in all tumors (Fig. 2B). Hypomethylation was most pronounced in T1 and T8 lymphomas and the least in T2 and T4 that had almost equal numbers of hypo- and hypermethylated DMCs (Fig. 2B). More stringent analysis of methylation changes using differentially methylated regions (DMRs; defined as ≥ 10% methylation change in the same direction in three consecutive cytosines in ≤ 100 bp, p < 0.05 (MWU)) confirmed the large-scale deregulation of methylation in all tumors. Notably, thousands of hyper-and especially hypomethylated DMRs were observed in all tumors in various genomic elements including promoters, enhancers, introns, exons, and repeats (Figs. 2C–G). Like in mouse hematologic malignancies^17–19^, the presence of hypo- rather than hypermethylated DMRs in promoters was a more frequent event in all tumors ranging from ~ 6,000 in T2 to ~ 20,000 in T8 (Fig. 2C). However, promoter hypermethylation was seen in all tumors and ranged from ~ 2,000 (T1) to 8,000 (T3). In some tumors ‒ T2 and T3 ‒ promoter hypo and hypermethylation were almost equal in frequency (Fig. 2C). Patterns of hypo- and hypermethylation changes didn’t appear to depend on tumor type since the most hypomethylated tumors ‒ T1 and T8 ‒ belonged to different PTCL subtypes (ALCL and PTCL-NOS, respectively).

While tumors varied in the frequency of hypo and hypermethylated DMRs in genomic elements, the trend remained similar within individual tumors. For example, the most hypomethylated tumors were T1 and T8, and hypomethylation was manifested across all genomic elements. Similarly, the ratio between hypo and hypermethylated DMRs was the smallest in T2, and that trend was seen across all genomic elements (Figs. 2C–G **and data not shown**).

Not surprisingly, the biggest ratio favoring hypo- over hypermethylated DMRs was observed in repetitive elements that are known to be prone to loss of methylation in tumors (Fig. 2G). Interestingly, as many as 767 hypo- and 567 hypermethylated DMRs were recurrently observed in all tumors tested (Figs. 2H, 2I, S4 **and Supporting Information 3**). We consider these high-frequency DMRs as representing the ‘Core PTCL Methylation Signature’. These DMRs were distributed in various genomic elements but especially in repeats and introns (Figs. 2I and S5). For example, *MPZL1* and *UCK2* loci uniformly hypermethylated in normal T-cells were hypomethylated in lymphomas with high frequency (Figs. 2J and S6). Similarly, *MIATNB* and *TXK* loci hypomethylated in normal T-cells gained methylation in all tested lymphomas. (Figs. 2J and S6). Among other notable genes with promoter hypermethylation were *LCK, CD101, PGGHG, RASSF3*, and *SLC39A2* genes, and with promoter hypomethylation *PTAFR, MPZL1, PRDM11*, *CTNND1, FLI1, GIT2, EML5, RBFOX1, KSR1*, and others (**Supporting Information 3**).

Further dissection of this signature may reveal methylation markers of PTCL associated with the disease initiation and progression, as well as genes driving the disease development.

### Analysis of gene expression in ALCL and PTCL-NOS

**Figure 3:**
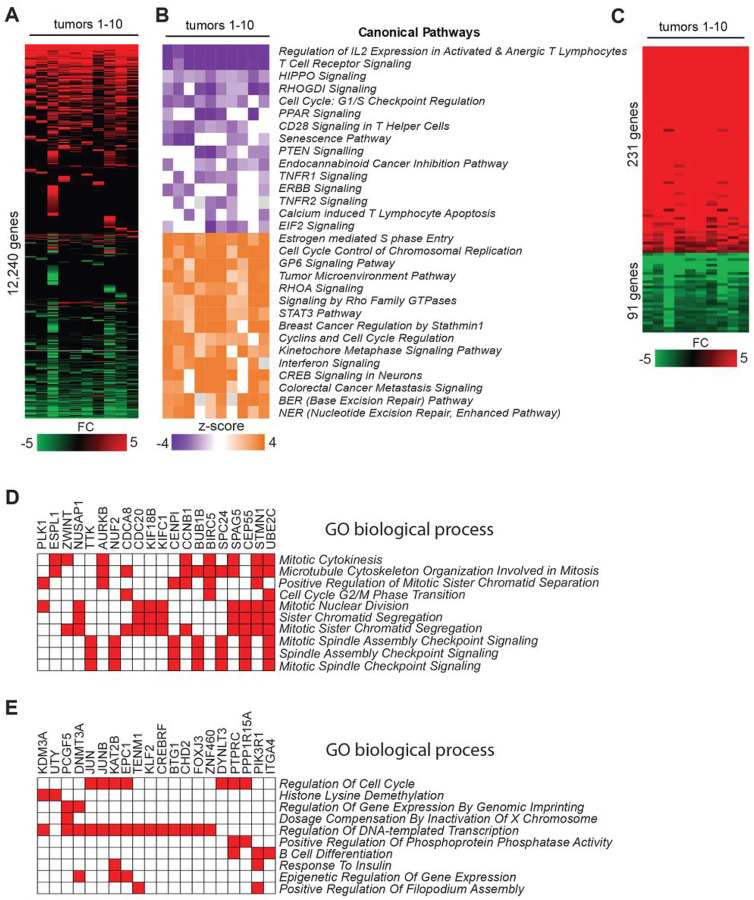
Gene expression signatures and deregulated pathways in PTCLs **(A)** Heat map showing fold change expression values of a subset of differentially expressed genes (DEGs; FC ≥ 1.5, p < 0.05 by DESeq2) in human T-cell lymphomas (n = 10) relative to control T-cells (n4) as analyzed by RNA-seq. A scale is shown below the heat map, in which green and red correspond to decrease and increase in expression, respectively. **(B)** Results of Ingenuity Pathway Analysis (IPA) performed on all DEGs in individual tumors. The top 15 up- and downregulated canonical pathways that were most consistently deregulated in analyzed human lymphomas are shown. **(C)** ‘Core PTCL Gene Expression Signature’ consisting of 231 up and 91 downregulated genes relative to the average of normal T-cells (n = 4) in all ten tested lymphomas as determined by RNA-seq data analysis. **(D)** Pathway enrichment analysis of upregulated genes in the ‘Core PTCL Gene Expression Signature’ in the category *GO Biological Process* by *EnrichR* (p < 0.05). **(E)** Pathway enrichment analysis of downregulated genes in the ‘Core PTCL Gene Expression Signature’ in the category *GO Biological Process* by *EnrichR* (p < 0.05).

To determine the extent to which methylation changes may contribute to deregulated transcriptome in PTCL, we next performed global gene expression profiling of PTCL samples using RNA-seq analysis. In addition to PTCL samples used for methylation analysis, we also included RNA isolated from three additional PTCL-NOS samples (T7, T9, T10) along with RNA from normal T-cells used for methylation analysis (Figs. 1, S1 and S2). These samples were subjected to RNA-seq analysis and the data were analyzed using *SeqMonk* software and DeSeq2 method to calculate differential expression. Our analysis revealed large-scale deregulation in transcriptomes of PTCL samples relative to normal T-cells manifested by findings that 12,240 genes were deregulated in at least one tumor relative to the averaged-out values of independent normal T-cell samples (Figs. 3A, S2 **and Supporting Information 4**).

Ingenuity Pathways Analysis (IPA) of differentially expressed genes [FC ≥ 1.5, *p*< 0.05 by DESeq2) in human T-cell lymphomas (n = 10) relative to control T-cells (n = 4)] revealed that all PTCLs had Inhibited Pathways related to *Regulation of IL-2 Expression in Activated and Anergic T Lymphocytes, T Cell Receptor Signaling*, and *HIPPO signaling* (Fig. 3B). Additional frequently suppressed pathways included *RHOGDI Signaling, Cell Cycle G1/S Checkpoint Regulation*, tumor suppressive *PTEN Signaling*, and others (Fig. 3B). In contrast, pathways frequently activated in all tested samples included *Estrogen-mediated S-phase Entry Cell Cycle Control of Chromosomal Replication*, *Signaling by Rho Family GTPases, and the STAT3* pathway. Additionally, there was a high frequency of activation in *Interferon* and *RHOA Signaling* pathways, along with other cancer-related pathways (Fig. 3B).

The expression of 231 genes was increased and the expression of 91 genes was decreased at least 2-fold in all ten PTCLs. We term these molecular changes as ‘Core Gene Expression Signature’ (Figs. 3C **and Supporting Information 5**). The TOP five genes overexpressed in all PTCL samples encode the following: phospholipase PLA2G2D, organic anion transporting polypeptide SLCO2B1, metalloprotease ADAMDEC1, complement protein C1QB, and receptor activity modifying protein RAMP3, with the average increase in expression over 500-fold. (**Supplementary Figs. S7 and Supporting Information 5**). None of these genes seem to have an established role in T-cell transformation and their deregulation can be a consequence of changes in signaling and epigenetic alterations in PTCL. GO enrichment analysis identified pathways linked to cell cycle regulation and in particular to *Cell Cycle G2/M Phase Transition, Mitotic Nuclear Division*, and *Sister Chromatid Segregation* (Fig. 3D). Analysis of genes not directly linked to the cycle revealed pathways related to *Complement Activation, Angiogenesis, Blood Coagulation*, and *Notch Pathway Signaling* (**Fig. S8**). ChIP enrichment analysis revealed the possible involvement of FOXM1, E2F1 and E2F4, SOX2, KLF4, and MYBL2 transcription factors in the pathogenesis of PTCL as they may play a role in the deregulation of the ‘Core PTCL Expression Signature’ (**Fig. S9**).

In all 10 PTCL samples, 91 genes exhibited a consistent decrease in expression, with at least a 2-fold reduction (Fig. 3C). Notably, the most frequently downregulated genes included *FGFBP2* (also known as *KSP37)*, teneurin *TENM1*, and a group of Y-linked genes such as *USP9Y, TTTY15*, and *UTY* (Figs. 3C and S7). Their role in lymphomagenesis remains unclear.

The GO enrichment analysis identified pathways linked to the *Cell cycle, Histone demethylation, epigenetic dysregulation*, and *B cell differentiation* likely occurring with the contribution of transcription factors FOXO1, FOXOM1, MYB, and others (Figs. 3E, S10 and S11).

Up-regulation of *TBX21* or *GATA3 and* their target genes (*EOMES*, *CXCR3, IL2RB, CCL3, IFN*_*Y*_, and *CCR4, IL18RA, CXCR7, IK* respectively) was previously shown to distinguish two subclasses of PTCL-NOS with distinct clinical outcomes^6,27^. To determine if any of the five samples included in our analysis belongs to a specific PTCL-NOS subtype, we analyzed RNA-seq expression patterns further. However, we did not see up-regulation of *TBX21* or *GATA3* when compared to normal controls in any of the five cases of PTCL-NOS perhaps because our analysis was limited by the small sample size (**Fig. S12**).

### DNA methylation modifiers are deregulated in human PTCL

**Figure 4:**
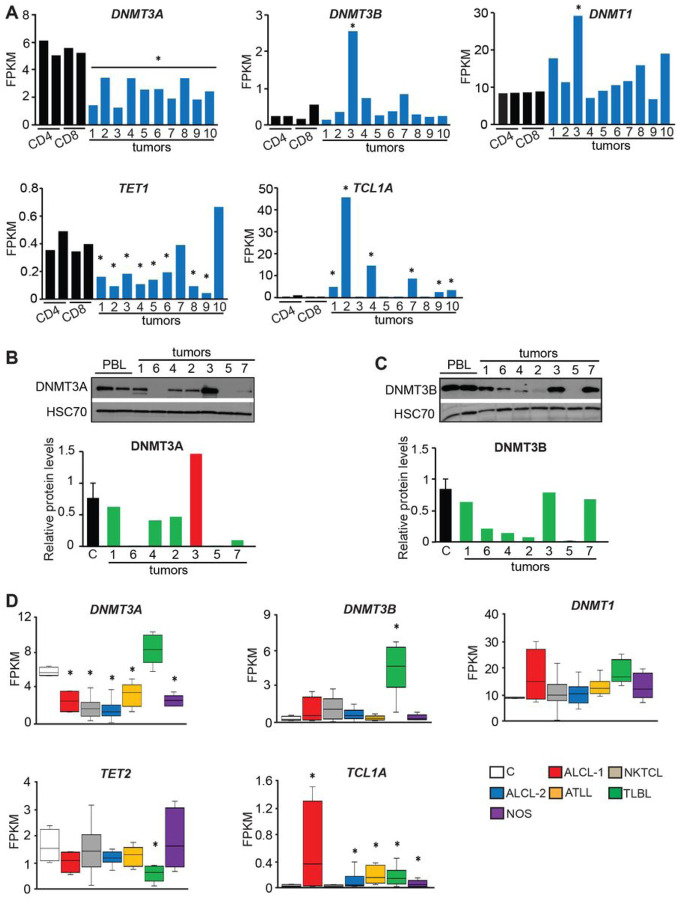
Expression of DNA methylation modifiers is deregulated in human PTCLs **(A)** Expression of *DNMT3A, DNMT3B, DNMT1, TET1*, and *TCL1A* in human PTCLs and control T-cells as analyzed by RNA-seq. Statistical analysis was performed by comparison of individual tumors to average values obtained from control T-cells (n = 4). The statistically significant differences (*p* < 0.05 by DESeq2) are indicated by *. **(B,C)** Immunoblot analysis of DNMT3A and DNMT3B protein levels in human PTCLs and two independent samples of peripheral blood lymphocytes (PBL). Bar graphs show relative DNMT3A and DNMT3B protein levels in individual lymphomas calculated from densitometric analysis of immunoblot signals relative to average values obtained from control PBLs. **(D)** *DNMT3A, DNMT3B, DNMT1, TET2*, and *TCL1A* expression as determined by RNA-seq analysis of normal human T-cells (C, n = 4), two sets of human Anaplastic Large Cell lymphomas (ALCL-1, n = 5 tumors T1-T5 profiled in this study; ALCL-2, n = 21 publicly available data), NKTCL T-cell lymphoma lines (NKTCL, n = 15), adult T-cell leukemia/lymphomas (ATLL, n = 8), T-lymphoblastic lymphomas (TLBL, n = 8) and PTCL-NOS (n = 5, tumors T6-T10 profiled in this study). The horizontal line represents the median, bounds of the box-like range of variation, and whiskers-min and max values. *p < 0.05 (by DESeq2).

To determine if levels of DNA methylation modifiers are deregulated in human PTCL, we further analyzed RNA-seq data and found that *DNMT3A* was significantly reduced in all tumors when compared to either CD4 + or CD8 + normal T-cells from peripheral blood (Fig. 4A). In contrast, *DNMT1* and *DNMT3B* were unchanged with only one ALCL tumor, T3, showing a significant increase in transcript levels (Fig. 4A). Analysis of DNA demethylases showed that *TET1* transcripts were low in all samples (FPKM < 1) but still significantly downregulated in the majority of tested tumors, whereas levels of *TET2* and *TET3* were unchanged relative to normal thymocytes (Figs. 4A and S13). Another protein that can affect DNA methylation is the TCL1A protein which was shown to inhibit DNA methyltransferases biochemically and affect global methylation in a mouse model of hematological malignancies^15^. We, therefore, analyzed its expression and found six out of ten analyzed tumors had significantly increased levels of *TCL1A* RNA relative to normal T-cells (Fig. 4A). In contrast, the expression of another member of the TCL family - *TCL1B ‒* that does not inhibit Dnmts 15 was unchanged in PTCL (**Fig. S13**).

To analyze protein levels, we next performed immunoblot analysis of DNMTs in a subset of PTCLs for which we had frozen tissues available (Tumors 1–7). Consistent with RNA-seq data analysis, DNMT3A was downregulated in most tumors relative to normal peripheral blood lymphocytes (Fig. 4B). Interestingly, we detected low protein levels of DNMT3B in T2, T4, T5, and T6 suggesting that despite unchanged mRNA levels, protein down-regulation may occur in primary PTCL (Fig. 4C). Whether DNMT3B levels are affected by TCL1A overexpression or the protein is down-regulated by other mechanisms, remains unclear. Unlike DNMT3A/B, DNMT1 levels did not seem to be changed in lymphomas (data not shown).

To determine whether deregulated expression of methylation modifiers is more broadly observed in PTCL subtypes, we next utilized publicly available data generated by RNA-seq analysis of 15 primary Natural killer/T-cell lymphomas (NKTCL), 21 ALCL (ALCL-2), eight Adult T-cell leukemia/lymphoma (ATLL), and eight T-lymphoblastic lymphomas (TLBL), and our five NOS and four ALCL (ALCL-1). While the expression of *DNMT1* was mostly unchanged, *DNMT3A* levels were significantly reduced across all PTCL subtypes except TLBL (Fig. 4D). Like *DNMT1, DNMT3B* expression was unchanged across most PTCL except for a significant increase in TLBL (Fig. 4D). The levels of *TET1* RNA showed a tendency for downregulation in a majority of analyzed PTCLs although it did not reach statistical significance, while *TET3* was mostly unchanged irrespective of tumor type (**Fig. S14**).

*TCL1A was* upregulated in a majority of PTCLs, whereas *TCL1B* was not significantly increased in any of the tested tumor subtypes (Figs. 4D, S14).

Altogether, this analysis revealed that several DNA methylation modifiers are deregulated in PTCL, with the downregulation of *DNMT3A* and *TET1* and up-regulation of *TCL1A* in most tested samples. These molecular changes, along with the downregulation of DNMT3B protein and genetic alterations found in these modifiers in a subset of PTCL as reported by others^28–3^, are the most likely reasons for the large-scale deregulation of PTCL methylomes we observed.

### Deregulated promoter methylation is associated with changes in gene expression

To determine the extent to which methylation changes in PTCL may affect transcription we further compared the data sets generated by WGBS and RNA-seq. First, we found that methylation changes also affected the expression of various repetitive elements, including DNA transposons, LTR and Non-LTR retrotransposons, and satellite repeats (**Fig. S15**). In a subset of repeat elements, such as *UCON80, LTR39*, and *LTR180*, hypomethylation mostly correlated with an increased expression. In contrast, hypomethylation of *UCON34* correlated with decreased, rather than increased expression (**Fig. S15**). However, we didn’t detect any consistent pattern of methylation changes, gene expression, or their correlation in examined tumors suggesting that repeats may not serve as good markers of disease development or progression.

**Figure 5:**
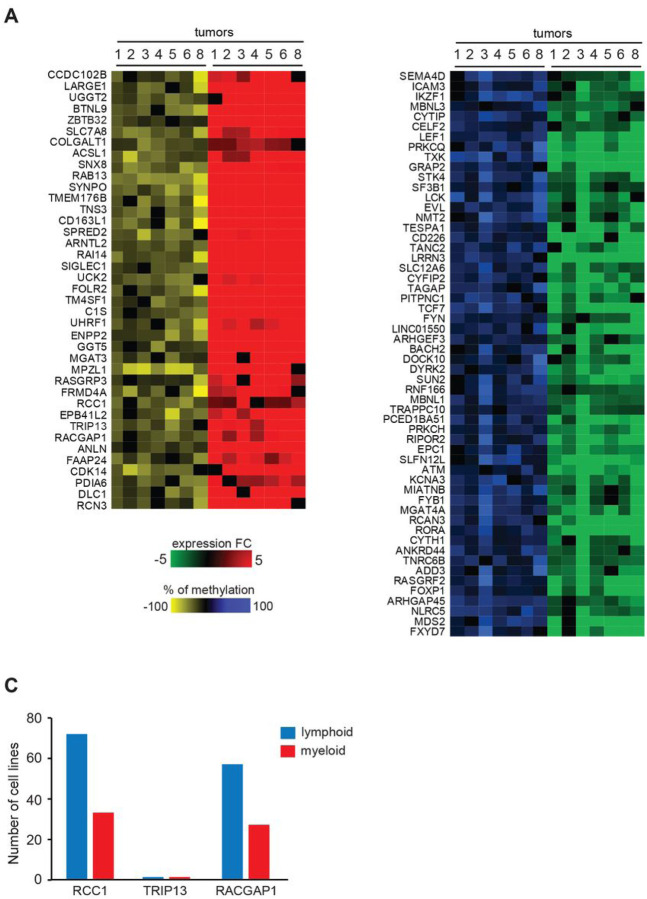
Promoter methylation is associated with changed gene expression for a subset of genes **(A)** *Left panel*. Heat map displaying 39 genes with hypomethylated promoters (presence of one or more hypomethylated DMRs in −1,500 bp to +500 bp) at a frequency at least six out of seven PTCLs and whose expression is increased relative to normal T-cell controls (≥ 2-fold change, p < 0.05) as determined by RNA-seq analysis. Numbers indicate individual tumors, the extent of hypomethylation is shown in yellow and hypermethylation in blue, while overexpression is shown in red and under expression in green. *Right panel*. Heat map displaying 56 genes with hypermethylated promoters (presence of one or more hypermethylated DMRs in −1,500 bp to +500 bp) at a frequency of at least six out of seven PTCLs and that were also under-expressed relative to normal T- cell controls (≥ 2-fold change and a p value < 0.05) as determined by RNA-seq analysis. **(B)** The number of lymphoid (blue) and myeloid (red) cell lines for which the knockout of *RCC1, TRIP13*, or *RACGAP1* genes was lethal. The numbers were determined using DepMap portal’s (Broad Institute) perturbation effects analysis and the gene effect was considered lethal if the expression log2(TPM+1) was ≥ −1.

Next, we examined gene expression changes that correlated with methylation changes in gene promoters at high frequency and found that 2,810 genes contained hypomethylated DMRs in 5 out of 7 tumors (**Supporting Information 6**). Out of those, 153 genes had increased expression in 5 out of 7 tumors (**Fig. S16**). *EnrichR* pathway enrichment analysis coupled with *Wiki Pathway* datasets revealed pathways regulating multiple cellular processes that are commonly upregulated in cancers, such as *VEGFA-VEGFR2 Signaling, PI3K-Akt signaling*, and *EGF/EGFR signaling (***Fig. S17**). Interestingly, several pathways related to Hippo signaling such as *Hippo-Merlin Signaling Dysregulation, Overview of leukocyte-intrinsic Hippo pathway Pathways Regulating Hippo* and *Hippo-Yap signaling pathway* raising the possibility that the deregulation of this pathway in T-cells could play a functional role in transformation. Promoters of 1,281 genes contained hypomethylated DMRs in at least 6 out of 7 tumors. Out of these, 39 genes were associated with increased expression (≥ 1.5) in PTCL suggesting that loss of methylation may have contributed to their deregulation (Fig. 5A).

Several genes from this group, such as *UHRF1, MPZL1, CDK14, RACGAP1, RAB13*, and others have oncogenic functions in various solid tumors, leukemias, and lymphomas either predicting poor prognosis, involved in tumor migration, metastasis, or maintenance^31–35^.

We identified 1,220 hypermethylated genes with the frequency of 5 out of 7 tumors out of which 74 are also downregulated in 5 out of 7 tumors (**Supporting Information 6 and Fig. S18**). Decreased expression of these genes was associated with the deregulation of various pathways including the *Wnt Signaling Pathway and Pluripotency* and *Pathways Regulating Hippo Signaling*. (**Fig. S19**). Promoters of 578 genes showed hypermethylated DMRs with a high frequency of 6 out of 7 PTCL. Out of these, the expression of 56 genes decreased by at least 1.5-fold. (Fig. 5A). Among these were genes with putative tumor suppressor function in different cancer types including *FOXP1, STK4,ATM*, and others^36–38^.

Interestingly, several genes with potential oncogenic functions, such as *SF3B1, FYN*, and *LCK* are also frequently downregulated but the physiological relevance of these changes for PTCL development remains unclear.

Our data show a relatively high number of recurrent methylation events observed in PTCL correlate with changes in gene transcription. Given that changes in expression affect many genes involved in various aspects of tumor biology, DNA methylation changes likely contribute to PTCL development in a causative way.

### Loss of DNA methylation correlates with up-regulation of genes critical for cancer cell proliferation

**Figure 6:**
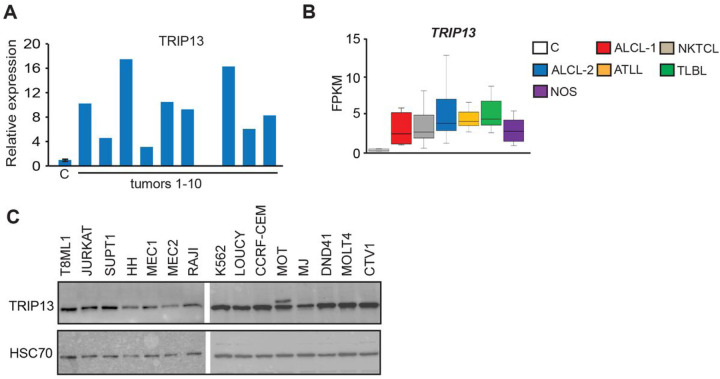
*TRIP13* is frequently up-regulated in primary human PTCL **(A)** Relative *TRIP13* expression as determined by RNA-seq. Data are presented as fold differences in FPKM values relative to the mean of normal T-cells (n = 2) obtained by RNA-seq analysis. **(B)** *TRIP13* expression as determined by RNA-seq analysis of normal human T-cells (C, n = 4), two sets of human Anaplastic Large Cell Lymphomas (ALCL-1, n = 5 tumors T1-T5 profiled in this study; ALCL-2, n = 21 publicly available data), Natural Killer T-cell Lymphomas (NKTCL, n = 15), Adult T-cell Leukemia/Lymphomas (ATLL, n = 8), T-Lymphoblastic Lymphomas (TLBL, n = 8) and PTCL-NOS (n = 5, tumors T6-T10 profiled in this study). The horizontal line represents the median, bounds of the box-like range of variation, and whiskers-min and max values. Pairwise comparisons between each tumor group and controls are statistically significant (*p* < 0.05 by DESeq2). **(C)** Immunoblot analysis of TRIP13 protein levels in human lymphomas and leukemia cell lines. T8ML-1 (PTCL-NOS); JURKAT (Acute T-cell leukemia); SUP-T1 (T-cell lymphoblastic lymphoma); HH (Cutaneous T-cell leukemia/lymphoma), MEC1, MEC2 (Chronic B-cell leukemia); RAJI (Burkitt’s lymphoma); K-562 (Chronic myelogenous leukemia); LOUCY, CCRF-CEM (Acute T-cell lymphoblastic leukemia); Mo T (Hairy T-cell leukemia); MJ (Cutaneous T- cell lymphoma); DND41 (T-acute lymphoblastic leukemia); MOLT4 (T lymphoblast cell); CTV-1 (T-ALL). HSC70 served as a loading control.

To further explore whether promoter hypomethylation may affect aspects of T-cell lymphomagenesis, we next focused on the analysis of 39 genes hypomethylated and overexpressed with high frequency in PTCL (Fig. 5A). To determine if any of these genes may play a role in PTCL maintenance, we utilized data from CRISPR knockout screens in the DepMap database (Broad Institute, (39, 40)) and investigated whether any of the genes affected the growth of hematologic cell lines. This search revealed that knockouts of most genes did not significantly impact the proliferation of hematological cell lines, as indicated by the gene effect scores ranging from − 0.75 to + 0.75 (Figs. 5B and S20). In contrast, the knockout of *RACGAP1* and *RCC1* genes was lethal to the majority of cell lines, and they were therefore classified as *Common essential* required for proliferation of cells in general. Interestingly, *TRIP13* (thyroid hormone receptor interactor 13) characterized by DepMap as *Strongly Selective* was critical for the maintenance of several cancer cell lines including B-cell lymphoma OCILY19 and Human eosinophilic leukemia EOL1. Because *TRIP13* is not a *Common essential* and therefore its targeting may be less toxic, we, therefore, focused on the analysis of this gene and sought to further explore its role in lymphomagenesis by determining its expression in primary TCL. Our analysis revealed that the gene was overexpressed in 9/10 PTCLs on average by ~ 10-fold relative to controls (Fig. 6A). Analysis of publicly available RNA-seq data revealed overexpression of *TRIP13* in ALCL, ATLL, NKTCL, TLBL and NOS (Fig. 6B).

We next performed immunoblot analysis of various cell lines to determine if the TRIP13 protein is present in hematologic cell lines. This analysis revealed that TRIP13 was expressed in T-cell lymphoma (T8ML-1, MJ, HH), T-cell leukemia (JURKAT, MOLT4, MOT, SUPT1, LOUCY, CCRF-CEM, DND-41) B-cell lymphoma (RAJI), B-cell leukemia (MEC-1, MEC-2) and myeloid leukemia (K562, CTV-1) cell lines (Fig. 6C). Altogether, our data show that TRIP13 is overexpressed in primary TCL and expressed in various hematologic cell lines and may be involved in their maintenance.

### TRIP13 downregulation inhibits the proliferation of malignant T-cells

**Figure 7:**
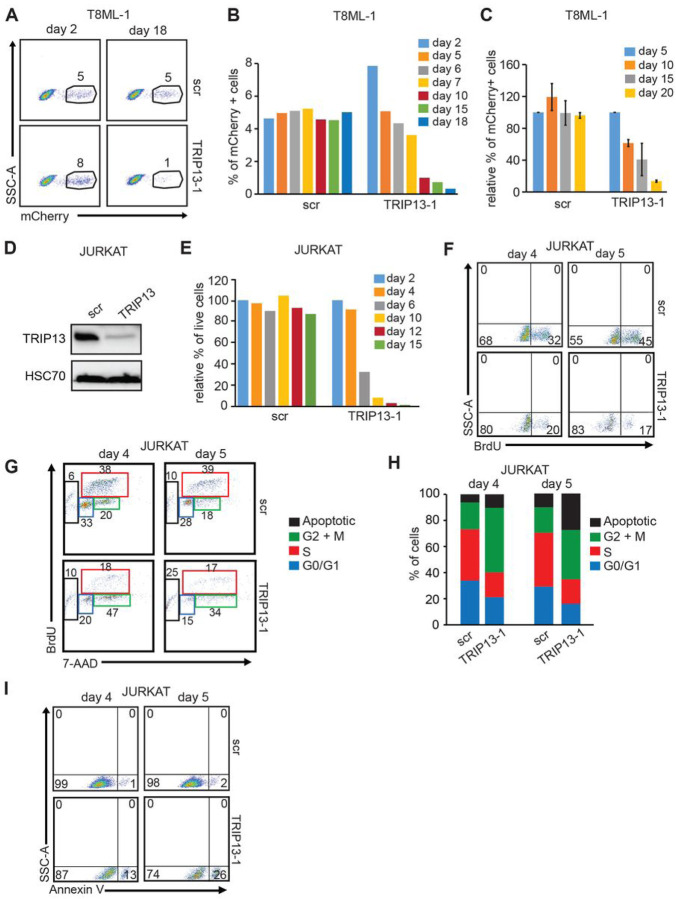
*TRIP13* downregulation results in impaired cellular proliferation of malignant T-cells **(A)** Representative examples of FACS diagrams indicating mCherry expression measured in unselected T8ML-1 cell line transduced with lentiviruses expressing shRNA against either *scrambled*(src) or *TRIP13* (shRNA-1). Data obtained at days 2 and 18 upon continuous culturing *in vitro are* shown. The percentage of mCherry-positive cells is shown above the gated population. **(B)** Percentage of mCherry-positive cells expressing shRNA-1 against either *scrambled* or *TRIP13*upon continuous culturing *in vitro* at indicated times as measured by FACS. **(C)** Relative mCherry expression in T8ML-1 cells transduced with lentivirus encoding shRNA-1 against *TRIP13 or scrambled*, measured by FACS 48 h after transduction (day 2) and 20 days later upon continuous culturing *in vitro*. The transduction efficiency, as measured by mCherry expression at day 2 was set to 100%, for both cells transduced with *scrambled* and *TRIP13* shRNAs. The values obtained for the percentage of mCherry-positive cells at each time point were plotted relative to the percentage at day 2. Data are presented as mean ±SEM (from three independent experiments) relative to *scrambled*, *p < 0.05. p values were calculated by a two-tailed *Student’s t-test*. **(D)** Immunoblot analysis of TRIP13 protein levels in JURKAT cells transduced with lentiviruses expressing either *scrambled or TRIP13* shRNAs. Cells were harvested and analyzed 72 h after transduction. HSC70 served as a loading control. **(E)** Relative percentage of live unselected JURKAT cells transduced with the indicated lentiviruses at 99% efficiency as measured by FACS analysis of mCherry expression co-expressed from the lentiviral constructs. Viability was determined as the percentage of cells in the *“live gat”* in forward scatter FACS diagrams at indicated times. The values obtained for the percentage of mCherry-positive cells at each time point were plotted relative to the percentage at day 2. **(F)** Representative examples of FACS diagrams obtained from BrdU incorporation assays of JURKAT cells transduced with lentivirus expressing shRNA against either *scrambled* or *TRIP13 as* determined at 4 and 5 after transduction. Cells were exposed to BrdU for 60 minutes, harvested and FACS analysis was used to evaluate the percentage of cells that incorporated BrdU. The percentage of positive cells in the FACS profile is shown within each plot. A representative example of two independent experiments is shown. **(G)** Representative examples of FACS diagrams obtained from cell cycle analysis of JURKAT cells transduced with lentivirus expressing shRNA against either *scrambled* or *TRIP13as* determined at 4 and 5 days after transduction. A combination of BrdU incorporation assay and staining with 7-ADD followed by FACS was used. **(H)** Percentage of cells in various phases of the cell cycle in JURKAT cells transduced with lentiviruses expressing shRNA against either *scrambled* or *TRIP13*, determined 4 and 5 days by BrdU incorporation assay coupled with 7-AAD staining and FACS-based analysis. **(I)** Representative examples of FACS diagrams obtained from Annexin V assays of JURKAT cells transduced with lentivirus expressing shRNA against either *scrambled* or *TRIP13* as determined at 4 and 5 days after transduction. The percentage of positive cells in the FACS profile is shown within each plot and indicates ongoing apoptosis.

The AAA ATPase TRIP13 (thyroid hormone receptor interactor 13) is known to participate in various regulatory steps related to the cell cycle, such as the mitotic spindle assembly checkpoint and meiotic recombination, as well as the DNA repair by immediate-early DNA damage sensing and ATM signaling activation^39^. To determine if TRIP13 is required for the proliferation of PTCL, we transduced a PTCL-NOS cell line, T8ML-1, with lentiviruses expressing shRNAs either targeting *scrambled* or *TRIP13* and co-expressing mCherry. Cells were cultured over twenty days and the percentage of mCherry positive cells was measured at different time points by FACS. The percentage of cells expressing *scrambled* shRNA remained relatively stable suggesting that expression of scrambled shRNA or mCherry did not affect the proliferation of cells (Figs. 7A and 7B). In contrast, the percentage of *TRIP13* shRNA-1 expressing cells was gradually reduced over time suggesting that the proliferation of T8ML-1 cells is impaired by *TRIP13* downregulation. (Figs. 7A and 7B). The proliferative defect was reproducible and was also seen using independent *TRIP13* shRNA-2 (Figs. 7C and S21). We next asked whether *TRIP13* knockdown can affect the proliferation of T-cell leukemia JURKAT cells. These cells could be routinely transduced to more than 99% efficiency thus allowing for direct cellular and molecular analysis (**Fig. S22**). As expected, both *TRIP1*3-specific shRNAs efficiently decreased RNA and protein levels of TRIP13 (Figs. 7D and S23). Similar to T8ML-1 cells, *TRIP13* knockdown resulted in impaired proliferation and reduced viability of JURKAT cells as determined by the decreased percentage of cells in *“live gat”* in FACS analysis and cell counts upon culturing over time (Figs. 7E **and data not shown**). *TRIP13* reduction resulted in decreased BrdU incorporation, G2-M arrest, increased Annexin V expression, and apoptosis (Figs. 7F–I). In contrast to downregulation, lentivirally mediated overexpression of C-terminally FLAG-tagged TRIP13 CDS from lentiviral backbone co-expressing fluorescent protein in T8ML-1 did not affect cell growth of T8ML-1 or JURKAT cells as the percentage of EGFP + cells remained relatively stable over time in both TRIP13 and control cells (**data not shown**). Altogether, these data suggest that the downregulation of *TRIP13* has antiproliferative effects by inducing G2-M arrest accompanied by apoptosis.

### Treatment of T8ML-1 cells with TRIP13 inhibitor DCZ0415 impairs proliferation and induces cell death

**Figure 8:**
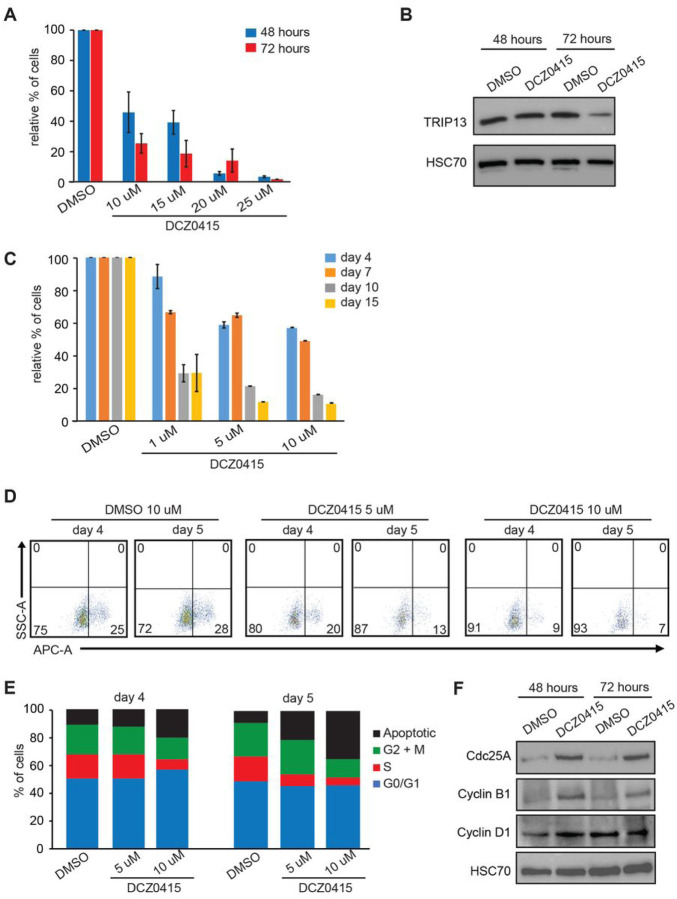
The treatment of T8ML-1 cells with DCZ0415 inhibits cellular growth by inducing G2-M arrest and cell death **(A)** T8ML-1 cells were treated with either DMSO (vehicle) or DCZ0415 at indicated concentrations and counted using Trypan Blue exclusion dye. Bar graphs represent the total cell counts of live cells after 48 hours (blue) or 72 hours (red) of continuous drug treatment. Each data point is the average of three measurements taken in parallel and bar graphs represent ±SEM. **(B)** Immunoblot analysis of TRIP13 protein levels in T8ML-1 cell line treated with either DMSO (vehicle) or DCZ0415 harvested and analyzed after 48 hours and 72 hours of continuous drug treatment. HSC70 served as a loading control. **(C)** T8ML-1 cells were treated with either DMSO (vehicle) or DCZ0415 at indicated concentrations for 15 days and counted using Trypan Blue exclusion dye. Bar graphs represent the relative % count of live cells after 4, 7, 10, and 15 days of continuous drug treatment. Each data point is the average of three measurements taken in parallel and bar graphs represent ±SEM. **(D)** Representative FACS diagrams of the BrdU incorporation assay of T8ML-1 cells treated with either DMSO, 5 or 10 μM of DCZ0415 after 4 and 5 days. Numbers in corners of FACS diagrams represent percentages obtained from quadrant statistics using FlowJo X software. **(E)** Percentage of cells in various phases of the cell cycle in T8ML-1 cells treated with either DMSO, 5 or 10 μM of DCZ0415 for 4 and 5 days. The numbers used to generate stacked graphs were obtained by analysis shown in **D** using the FACS data obtained from BrdU incorporation assay coupled with 7-AAD staining and analyzed by FlowJo X software. **(F)** Immunoblot analysis of Cdc25A, Cyclin B1,and Cyclin D1 protein levels in T8ML-1 cell line treated with either DMSO (vehicle) or DCZ0415 harvested and analyzed after 48 hours and 72 hours of continuous drug treatment. HSC70 served as a loading control.

To test if targeting TRIP13 may have therapeutic potential, we next treated lymphoma cell lines with DCZ0415, a TRIP13-specific inhibitor^40^. The treatment of T8ML-1 cells with various concentrations of DCZ0415 (10–25 μM) showed dose-dependent cell death with EC50 values of 10.0 μM and 5.5 μM upon 2- and 3-day treatment, respectively (Figs. 8A and S24). The cell treatment was accompanied by the downregulation of endogenous TRIP13 protein suggesting not only that DCZ0415 inhibits this protein but also contributes to its downregulation accompanied by impaired proliferation and cell death (Fig. 8B). When T8ML-1 cells were treated longer, even lower DCZ0415 concentration effectively impaired cellular proliferation. For example, a 14-day treatment of T8ML-1 cells severely reduced viability at 5 μM and 10 μM DCZ0415 concentrations (Fig. 8C). Even at concentrations as low as 1 μM, DCZ0415 reduced cellular viability by 50%. Like with shRNA-mediated *TRIP13* downregulation, the treatment with DCZ0415 inhibitor impaired cell proliferation by reducing BrdU incorporation, inducing G2-M arrest, and cell death (Figs. 8D and 8E). Consistently with G2-M arrest, the levels of G2-M proteins - Cdc25A and Cyclin B1 ‒ were elevated upon the drug treatment, whereas G1-S Cyclin D1 was downregulated.

Altogether, our data show that targeting TRIP13 may be beneficial in treating PTCL.

## Discussion

The goal of our studies was to better understand the molecular landscape of PTCL with a focus on recurrent molecular changes and identification of putative genes driving lymphomagenesis. Using high-resolution methylation of seven PTCLs and gene expression profiling of ten PTCLs coupled with bioinformatic analysis of previously published data and functional studies, we made several interesting observations in this study.

First, hypomethylation of the PTCL genome is more frequent than hypermethylation regardless of the genomic elements or tumor types. These data are consistent with the numerous studies in mouse models that demonstrated that hypomethylation in T-cell malignancies is more frequent than hypermethylation^17,20,41^ Furthermore, methylation patterns in tumors are heterogonous with large differences and only a few common features. Some studies in hematological malignancies found tumor methylation patterns to have little to no cancer specificity. For example, based on the profiling of Chronic Lymphocytic Leukemia (CLL) it was proposed that tumor patterns are reflections of the methylation status of the cell of origin, rather than being truly cancer-specific as most previously reported tumor-specific methylation events are normally present in non-malignant B-cells^42^. Similarly, recently identified DNA methylation patterns of ALCL were found to be similar to thymic progenitor cells^24^. Due to the tremendous methylation heterogeneity we observed across PTCLs, the possibility that PTCLs reflect methylation patterns of the cell of origin appears unlikely. Rather, methylation differences between normal and malignant T-cells appeared to be a consequence of at least three molecular events we observed with high-frequency ‒ downregulation of DNMT3A on the transcript level, downregulation of DNMT3B protein, and up-regulation of TCL-1 protein that may biochemically inhibit DNMT functions. These molecular changes are likely responsible for the ‘Core PTCL Methylation Signature’ consisting of 767 hypo- and 567 hypermethylated DMRs that were observed across various genomic elements in all seven analyzed PTCLs. This signature had no overlap with the 12 gene signature (hyper - *BCL11B, CD5, CXCR6, GIMAP7, LTA, SEPT9, UBAC2, UXS1*);hypo -*ADARB1, NFIC, NR1H3, ST3GAL3)* observed in Hepatosplenic T-cell lymphoma^25^. Interestingly, we observed hypermethylation of *LCK*, previously shown to be hypermethylated and repressed in ALCL and PTCL samples^24^. Although not part of the ‘Core PTCL Methylation Signature’, we also observed hypermethylation of *LEF1* and *TCF7*, but not *BCL11B* reported previously in PTCLs^24^. Thus, *LCK* promoter hypermethylation and to a lesser degree *LEF1* and *TCF7* belong to the most frequent molecular changes characterizing PTCLs. In addition to methylation signatures, we also identified the ‘Core PTCL Expression Signature’ consisting of 231 genes that were up- and 91 genes that were downregulated in all 10 tested tumors. Not surprisingly, at least 48 genes in this signature were associated with the cell cycle. Additional genes in the signature were related to complement activation, blood coagulation, and Notch pathway signaling. Several genes of the ‘Core PTCL Expression Signature’ are less characterized but their recurrent deregulation suggests a potential association with tumor development and have the potential to serve as biomarkers. Among them is *TEDC2* (also known as *C16orf59)* whose increased expression is associated with poor prognosis of lung adenocarcinoma^43^. Overexpression of *ZWINT* predicts poor prognosis and promotes the proliferation of hepatocellular carcinoma^44^. Increased *C10orf10* levels (DEPP Autophagy Regulator 1, *DEPP1)* correlated with the shorter survival time of patients with primary gliomas^45^.

Second, we identified recurrent methylation events in promoters that were associated with the changes in gene expression. Namely, 39 genes upregulated in PTCL whose promoters were hypomethylated, and 56 genes repressed in tumors whose promoters were hypermethylated. Importantly, several oncogenes and tumor suppressor genes were identified in these signatures. For example, the hypomethylated and overexpressed gene *UHRF1* has oncogenic functions promoting tumorigenesis through the silencing of DNA repair genes and inhibiting apoptosis in various cancers^32^. *UHRF1* is overexpressed in T-cell ALL and its knockdown reduces c-MYC expression and viability in these malignancies^33^. Other genes hypomethylated and overexpressed in PTCL - *RAB13, MPZL1, CDK14- were* implicated in tumor progression of several different tumors including B-cell lymphomas, and glioblastomas, lung and ovarian^34,35,46,47^. Among genes, we detected to be hypermethylated and silenced in PTCL are major tumor suppressors, e.g. *ATM*, which plays a critical role in DNA repair and whose functions are often compromised in PTCL through acquired genetic alterations^48^. Another hypermethylated and silenced gene in PTCL - serine/threonine-protein kinase 4 (*STK4)* ‒ regulates cell differentiation and apoptosis and is the tumor suppressor in hepatocellular carcinoma, breast cancer, and lymphoma^49^. We also detected somewhat puzzling hypermethylation and silencing of genes that would be predicted to be oncogenes, rather than tumor suppressors, such as *SF3B1, LCK, FOXP1, FYN*^50,51^. Given the complexities of gene functions and the large-scale-deregulation of signaling networks in cancer, it is not surprising that some genes normally promoting tumorigenesis would be silenced. Alternatively, their effects on tumorigenesis would be context-dependent. For example, loss of proto-oncogene *LCK* accelerates CLL development in mice suggesting the tumor suppressor role for this gene^52^. The specific roles of genes whose expression changes coincided with methylation alterations remains to be worked out. However, our data strongly suggests that both DNA hypo- and hypermethylation affect the expression of genes that are important in various aspects of T-cell tumorigenesis and at least some of them are likely to contribute to PTCL development.

Third, we found that genetic and pharmacological inhibition of TRIP13 impaired the proliferation of T-cell malignant cell lines by inducing G2-M arrest and apoptosis. Interestingly, we identified mouse *TRIP13 to* be hypomethylated and overexpressed in mouse T-cell lymphomas^21^. Our findings collectively underscore the critical role of DNA methylation in regulating *TRIP13*, as well as its significant involvement in the development and/or maintenance of TCL. This positions *TRIP13* as a potential key oncogene in PTCL. Reinforcing this hypothesis, similar conclusions have been drawn from research conducted on various solid tumors, where the influence of *TRIP13* on cancer progression has been similarly observed. For example, *TRIP13* is highly expressed in Glioblastoma and its inhibition impaired the proliferation, migration, and invasion of tumor cells by regulating c-MYC stability^53^. TRIP13 promotes metastasis of colorectal cancer and its inhibition decreased cell proliferation *in vitro* and tumor formation *in vivo* of colorectal carcinoma cell lines^40–54^.

Our functional assays demonstrate that promoter hypomethylation is important even in human lymphomagenesis, at least in its maintenance. Whether it plays a role in initiation and progression remains to be tested.

It’s important to note certain limitations in our study. Although our use of WGBS allowed for a more comprehensive analysis of the methylation landscape of PTCLs than previous studies^24,25^, our research was limited to seven samples. This limitation might impact the generalizability of our findings in the broader context of PTCL research. Furthermore, while we performed RNA-seq gene-expression analysis on 10 PTCL samples, only seven of those lymphomas were profiled for DNA methylation alterations due to the unavailability of DNA. Altogether, this limited our ability to address tumor-specific differences between ALCL (T1–4), AITL (T5), and PTCL-NOS (T5–10). Thus, by and large, we did not attempt to dissect the molecular differences within tumor subtypes as such analysis would result in a large number of false-positive and negative alterations and therefore provide substantially skewed views of molecular landscapes. Rather, our focus was on methylation and gene expression landscapes of PTCLs analyzed as one single tumor entity in an attempt to identify potential molecular events involved in the pathogenesis of these malignancies. In one analysis attempting to classify PTCL-NOS into *TBX21* or *GATA3* subgroups, we were unable to do so either using their gene expression values or readout of their target genes. While this might be due to the limited sample size, it is also possible that such classification of PTCL-NOS, which was primarily based on microarray results, may not be easily seen by RNA-seq analysis or be less pronounced than previously thought.

Our future study will also address the relationships of methylation and gene expression changes observed in this study. However, given the large number of genes with demonstrated biological effects in T-cell biology, our study clearly points to a causative relationship of deregulated methylation in PTCL development.

## Methods

### Clinical samples and data sources

The study included in total two normal human purified CD4 + T-cells (84200–1.0/13122) and normal human purified CD8 + T-cells (84300–1.0/13083) obtained from Precision Medicine. These samples were used for methylation analysis by WGBS and gene expression analysis by RNA-seq. We also used seven frozen PTCL tissue samples and one normal lymph node sample obtained from the NCI Cooperative Human Tissue Network (CHTN). Other investigators may have received samples from the same tissue specimens. PTCL samples were used for the isolation of DNA (for WGBS), RNA (RNA-seq and real-time qRT-PCR), and proteins (immunoblots). The frozen lymph node was used for protein isolation and served as a control for immunoblots. Additional three total RNA samples isolated from PTCL tumors and one paired DNA sample were acquired from Origene (Rockville, MD, USA). Methylation and gene expression analyses were done on paired DNA-RNA samples except for two RNA samples for which DNA or tissue was not available. The Institutional Review Board of the University of Nebraska Medical Center approved the use of these samples. The sample sets are described in this section, in Figures S1 and S3. For data analysis, we utilized several data sets available online. For the methylation analysis - in addition to normal human T-cell controls generated in this study - we also used publicly available WGBS data on CD3 + and CD4 + T-cells obtained from GEO [GSM1186660, GSM2103005, GSM2103006, GSM2103007]^55^. For gene expression analysis publicly available data for human ALCL (SRA accession numbers SRR1522989, SRR1522990, SRR1522992, SRR1522994, SRR1522996, SRR1522997, SRR1522998, SRR1522999, SRR1523000, SRR1523001, SRR1523002, SRR1523003, SRR1523004, SRR1523005, SRR1523006, SRR1523007, SRR1523008, SRR1523009, SRR1523010, SRR1523011, SRR1523012), ATLL - GSE143986 (SRR10918774, SRR10918775, SRR10918776, SRR10918777, SRR10918778, SRR10918779, SRR10918780, SRR10918781)^56^ NKTCL (SRR1648322, SRR1648328, SRR1648327, SRR1648323, SRR1648332, SRR1648329, SRR1648315, SRR1648333, SRR1648324, SRR1648318, SRR1648326, SRR1648330, SRR1648321, SRR1648331, SRR1648325) and TLBL -GSE109231 (SRR6495843, SRR6495840, SRR6495836, SRR6495842, SRR6495844, SRR6495835, SRR6495837, SRR6495841^57^ were used. (https://bmccancer.biomedcentral.com/articles/10.1186/s12885-018-4304-y)^55^ (**Supporting Information 7**).

### Plasmid DNA constructs

Lentiviral vectors pLV[shRNA]-mCherry-U6 > scrambled [shRNA#1] with a target sequence of CCTAAGGTTAAGTCGCCCTCG, pLV[shRNA]-mCherry-U6 > hTRIP13[shRNA#1] with a target sequence of GCTACTCAACAGACATAATAT, pLV[shRNA]-mCherry-U6 > hTRIP13[shRNA#2], with a target sequence of GATGAAGTGTCAGATCATATA, pLV-EF1 A-mCherry and pLV-EF1A hTRIP13 −3XFLAG- P2A-mCherry were obtained from VectorBuilder.

### Cell lines and lentiviral production

Human peripheral T-cell lymphoma cell line T8ML-1 was a generous gift from Dr. Hiroshi Fujiwara (Ehime University, Japan). Human acute T-cell leukemia cell line JURKAT (#TIB-152) was obtained from the American Type Culture Collection (ATCC). Lenti-XTM 293T Cell Line was purchased from Takara (#632180, Clontech). The cell lines was periodically checked for karyotype and tested for mycoplasma contamination. Only early passages were used.

Cells were maintained in DMEM or RPMI 1640 (Invitrogen) containing 10% fetal bovine serum. Cell lines were cultured at 37°C in a humidified 5% CO2 atmosphere and were passaged according to recommendations.

To generate lentiviruses, 293T cells were seeded in a 100 mm tissue culture plate to obtain ~ 80–90% confluence and transfected with plasmid DNA and packaging plasmids psPAX2 and pMD2.G at a ratio 1:0.65:0.35 using 70 μg of polyethylenimine (PEI) (Polysciences). Viruses were collected 48 −96 h post-transfection. Transduction was performed as described previously 17 using Polybrene Infection / Transfection Reagent (Sigma). The efficiency of transduction was determined by measuring the percentage of mCherry-positive cells by FACS.

### FACS, BrdU incorporation, and apoptosis assays

For shRNA experiments, the growth of T8ML-1 or JURKAT cells transduced with lentiviruses was monitored by FACS over 2–20 days. The maximum percentage of mCherry-expressing cells was typically observed 72 hours post-transduction. In some experiments, all subsequent mCherry data points were normalized to this time point and expressed as a relative percentage of the initial time point. EGFP was measured periodically by flow cytometry on the LSRII available at the University of Nebraska flow cytometry core facility. In JURKAT cells, where we achieved + 99% transduction efficiency, the growth of cells was evaluated by Trypan blue staining and cell counting at different time points. Viability was also evaluated by scoring percentages of cells in the “Live gate” of FACS diagrams obtained using cells at different timepoints. For in vitro BrdU labeling, BrdU at a concentration of 0.01 mM was added 110 min before harvests of cells. BrdU-positive cells were quantified using anti-BrdU-FITC (BrdU- Flow Kit, BD Biosciences PharMingen) as described by the manufacturer. For cell cycle analysis, 7-ADD was added to the samples. For analysis of apoptosis, cells were stained with allophycocyanin-conjugated annexin V antibody and analyzed by FACS according to the manufacturer’s recommendations (eBioscience). FACS analysis was done using the BD Accuri C6 Plus Flow Cytometer.

### WGBS and bioinformatics analysis

Genomic DNA from PTCL samples was isolated using QIAamp DNA Mini Kit (Qiagen).

The WGBS libraries using DNA from normal human CD4 + and CD8 + purified T-cells and seven PTCLs were prepared and sequenced on an Illumina NovaSeq6000 sequencer using 150 bp long paired-end reads by Novogene, USA. Quality checks, trimming, filtering, alignment of reads to the UCSC hg38 reference genome, and methylation calling were performed with Bismark software^58^. Only CpG sites with a minimum sequencing depth 5x were included in the analysis. Differential methylation was calculated by comparison of individual tumors to the control consisting of methylation data obtained from five normal human T-cells - CD3, CD4_1, CD4_2, CD4_3, and CD8 - averaged out by Metilene.

Differentially Methylated Regions (DMRs) were determined by *Metilene*^59^ and defined based on the average of a minimum of three consecutive DMCs with methylation change of ≥ 10% in the same direction with p values < 0.05 (as determined by MWU test). The maximal base pair cut-off for a distance between consecutive DMCs in DMR was set to 100 bp. Annotation of methylated CpGs and DMRs to promoters, gene bodies, enhancers, and repeats was performed using bedtools intersect. Chromosomal coordinates of TSS, gene bodies, and repeats were acquired from the USCS Table browser. Enhancer coordinates identified in CD4 + CD8 + cells and thymus cells were obtained from Enhancer atlas^60^. Promoter was defined as 1,500 bp upstream to 500 bp downstream of the TSS.

Methylation scores were visualized with the Integrated Genome Browser (IGB)^61^. Scatter plots of methylation score were generated in RStudio v1.1.4.6 using package gplots. Genome-wide Pearson correlation analysis of CpG sites was performed using deepTools package multi bigwig summary and plot Correlation^62^.

### Immunoblotting

Frozen tissue was cut on a glass plate on dry ice and 20–50 mg of tissue was placed into a round bottom Eppendorf tube. 20 μL/mg of ice-cold RIPA buffer (50 mM Tris-HCl (pH 7.4), 150 mM NaCl, 1 mM EDTA, 1% NP-40, 1% Na-deoxycholate, 0.1% SDS, sterile-filtered) along with protease inhibitor cocktail and sodium orthovanadate was added and tissue was homogenized with an electric homogenizer on ice. After 20 min incubation on ice, samples were centrifuged at 13,000 x g for 20 minutes at 4°C. The supernatant containing the soluble protein was collected into a new tube kept on the ice. Immunoblots were performed as previously described 17, using the following antibodies detecting: DNMT3A (cat.# SC-20703, Santa Cruz; dilution 1:1000), DNMT3B (cat.# PA1–884, Thermo Fisher; dilution 1:1000), TRIP13 (cat.# TA809737S, Origene; dilution 1:1000), Cyclin B1 (cat.# 4138, Cell Signaling; dilution 1:1000), Cyclin D1 (cat.# 2978, Cell Signaling; dilution 1:1000), Cdc25A (cat.# SC-97, Santa Cruz; dilution 1:500) and HSC70 (cat.# SC-7298, Santa Cruz; dilution 1:10000). Relative densitometric values of protein levels were calculated using ImageJ software.

### RNA isolation, RNA-seq, and bioinformatics analysis

Total RNA from frozen tumor tissues was isolated using the TRIzol reagent (Invitrogen) and repurified using an RNAeasy kit (Qiagen). Library generation and sequencing on NovaSeq 6000 platform using paired-end 150 bp runs was performed by Novogene, USA. Tissue samples from seven Peripheral T-cell lymphomas were provided by the NCI Cooperative Human Tissue Network (CHTN). Other investigators may have received samples from the same tissue specimens. Three total RNA samples isolated from PTCL tumors were acquired from Origene (Rockville, MD, USA). The Institutional Review Board of the University of Nebraska Medical Center approved the use of these samples. The sample description is summarized in Supplementary Fig. 2.

Publicly available data for human ALCL, ATLL, NKTCL, and TLBL were obtained from GEO^55^ and SRR (accession numbers listed in Supporting Information 7). Trimmed sequencing data were first aligned to *Homo sapiens* UCSC hg38 reference genome using STAR aligner. RNA-seq data with minimum mapped quality 50 were quantified using the RNA-seq quantification pipeline in SeqMonk software (http://www.bioinformatics.babraham.ac.uk/projects/seqmonk/). DESeq2 was used to calculate differential expression. For differentially expressed genes, only genes with a fold change ≥ 1.5 and a p value < 0.05 were considered significant. Ingenuity pathway analysis (Qiagen)^63^ was used to analyze activated and decreased core signaling pathways for differentially expressed genes. Activated and inhibited pathways (Z-score > 1.5, p < 0.05) identified in individual PTCLs are shown in Fig. 3B. The top subcategories obtained in Physiological System, Development, and Functions are displayed (p < 0.05, for all subcategories).

### TRIP13 drug treatment, cell counting, and molecular assays

DCZ0415, an inhibitor of TRIP13, was obtained from (MedChem, Cat. #: HY-130603, Monmouth Junction, NJ, USA) and dissolved in DMSO. T8ML-1 cells were subjected to treatments with either DMSO (serving as the vehicle control) or the DCZ0415 at concentrations ranging from 1 to 25 μM per 1 mL of culture. The cell viability was assessed using Trypan Blue exclusion dye cell counts at different time points after the drug addition. EC50 values were determined by assessing the drug concentration that corresponds to 50% cell viability and were analyzed by AAT Bioquest Software. For the analysis of TRIP13 protein levels upon the treatment, 1 × 10^8^ T8ML-1 cells were treated with DMSO or 20 μM DCZ0415 per 1 mL of culture, and protein lysates were made similarly as described above. BrdU assays and apoptosis were done as described above.

### Real-time qRT-PCR

Two micrograms of RNA were reverse transcribed with the superscript III Reverse transcriptase (Thermo Fisher) using oligo (dT) primers. Real-time qRT-PCR was performed with the iQ^™^ SYBR^®^ Green Supermix (Bio-Rad) on a CFX96 Touch^™^ Real-Time PCR Detection System (Bio-Rad). Fast PCR cycling conditions were used (95°C for 3 min, 40 cycles (95°C for 10 s, 58–63.5°C for 30 s)), followed by a dissociation curve analysis. All qPCR measurements were performed in duplicate reactions and normalized to the expression of the housekeeping gene (*β-actin)*. In parallel, no-RT controls were amplified to rule out the presence of contaminating genomic DNA (**Supporting Information 8**).

### Statistical analysis

The statistical significance of means ± SEM was evaluated using the two-tailed Student’s t-test. For all statistical analyses p values < 0.05 were considered significant.

## Data Availability

All relevant data are available from the corresponding author upon reasonable request. The WGBS and RNA-seq are deposited at the NCBI Gene Expression Omnibus database [wgbs_accesion, GSE154451]^55^.

## Abbreviations and nomenclature

7-ADD: 7-aminoactinomycin D
AAA: ATPases Associated with diverse cellular Activities
ADAMDEC1: A disintegrin and metalloproteinase domain-like protein decysin-1
ADARB1: Adenosine deaminase RNA specific B1
AITL: Angioimmunoblastic T-cell Lymphoma
ALCL: Anaplastic Large Cell Lymphomas
ALK: Anaplastic Lymphoma Kinase
ATCC: American Type Culture Collection
ATM: Ataxia-Telangiesctasia Mutated
BCL11B: B-cell leukemia/lymphoma 11B
BCLs: B-cell Lymphomas
BrdU: Bromodeoxyuridine
C1QB: Complement C1q B Chain
CCL3: C-C motif chemokine ligand 3
CCR4: C-C motif chemokine receptor 4
CD: Cluster of differentiation
CD101: Cluster of Differentiation 101
CDK14: Cyclin dependent kinase 14
CDS: Coding sequence
CGs: CpG Dinucleotides
CHOEP: Cyclophosphamide, Doxorubicin, Vincristine, Etoposide, Prednisone
CHOP: Cyclophosphamide, Doxorubicin, Vincristine, Prednisone
CHTN: Cooperative Human Tissue Network
CLL: Chronic Lymphocytic Leukemia
CTNND1: Delta 1 catenin
CXCR3: C-X-C Motif Chemokine Receptor 3
CXCR6: C-X-C motif chemokine receptor 6
CXCR7: C-X-C chemokine receptor type 7
DEPP1: Decidual protein induced by progesterone-1
DESeq: Differential gene expression analysis based on the negative binomial distribution
DMCs: Differentially Methylated Cytosines
DMEM: Dulbecco’s Modified Eagle Medium
DMRs: Differentially Methylated Regions
DNMTs: DNA methyltransferases
E2F1: E2F Transcription Factor 1
E2F4: E2F Transcription Factor 4
EGF: Epidermal growth factor
EGFP: Enhanced green fluorescent protein
EGFR: Epidermal Growth Factor Receptor
EML5: Echinoderm Microtubule Associated Protein Like 5
EOL1: Human eosinophilic leukemia
EOMES: Eomesodermin
FGFBP2: Fibroblast growth factor binding protein 2
FITC: Fluorescein isothiocyanate
FLI1: Friend leukemia integration 1
FOXO1: Forkhead box O1
FOXOM1: Forkhead box M1
FOXP1: Forkhead box P1
FPKM: Fragments Per Kilobase of transcript per Million mapped read
FYN: FYN proto-oncogene/ Src family tyrosine-protein kinase
GATA3: GATA Binding Protein 3
GEO: Gene Expression Omnibus
GIMAP7: GTPase, IMAP family member 7
GIT2: GTPase-Activating Protein 2
GTPases: Guanosine triphosphatase
IFN_Y_: Interferon Gamma
IGB: Integrated Genome Browser
IK: Interferon inhibiting cytokine factor
IL18RA: interleukin-18 receptor 1
IL-2: Interleukin-2
IL2RB: Interleukin-2 receptor subunit beta
IL-7: Interleukin-7
IPA: Ingenuity Pathways Analysis
KLF4: Kruppel-like factor 4
KSP37: Killer-specific secretory protein of 37 kDa
KSR1: Kinase Suppressor of RAS 1
LCK: Lymphocyte-specific protein tyrosine kinase
LEF1: Lymphoid enhancer binding factor 1
LTA: Lymphotoxin Alpha
LTR: Long terminal repeats
MIATNB: Myocardial infarction associated transcript neighbor
MPZL1: Myelin protein zero like 1
MTCLs: Mature T-cell lymphoma
MWU: Mann‒Whitney U test
MYB: Myeloblastosis proto-oncogene protein
MYBL2: MYB Proto-Oncogene Like 2
NFIC: Nuclear factor I C
NHLs: Non-Hodgkin’s Lymphomas
NKTCL: Natural Killer/T-cell Lymphomas
NR1H3: Nuclear Receptor Subfamily 1 Group H Member 3
PEI: Polyethylenimine
PGGHG: Protein-Glucosylgalactosylhydroxylysine Glucosidase
PI3K: Phosphoinositide-3-kinase
PLA2G2D: Phospholipase A2 group IID
PRDM11: PR/SET domain 11
PTAFR: Platelet-activating factor receptor
PTCL-NOS: PTCL-not Otherwise Specified
PTCLs: Peripheral T-cell lymphomas
PTEN: Phosphatase and Tensin homolog
RAB13: Ras-Related Protein 13
RACGAP1: Rac GTPase activating protein 1
RAMP3: Receptor Activity Modifying Protein 3
RASSF3: Ras Association Domain Family Member 3
RBFOX1: RNA Binding Fox-1 Homolog 1
RCC1: Regulator of chromosome condensation 1
RHOA: Ras Homolog Family Member A
RHOGDI: Rho GDP-dissociation inhibitor
RIPA: Radioimmunoprecipitation Assay
SEPT9: Septin 9
SF3B1: Splicing Factor 3b Subunit 1
SLC39A2: Solute carrier family 39 member 2
SLCO2B1: Solute carrier organic anion transporter family member 2B1
SOX2: SRY-Box Transcription Factor 2
ST3GAL3: ST3 Beta-Galactoside Alpha-2,3-Sialyltransferase 3
STAR: Spliced Transcripts Alignment to a Reference
STAT3: Signal transducer and activator of transcription 3
STK4: Serine/Threonine-Protein Kinase 4
TBX21: T-box transcription factor
TCF7: Transcription Factor 7
TCL: T-cell lymphoma
TCL1A: T-cell leukemia/lymphoma protein 1A
TCR: T-cell Receptor
TEDC2: Tubulin epsilon and delta complex 2
TENM1: Teneurin transmembrane protein 1
TET: Ten-elevan translocation
TET1: Tet Methylcytosine Dioxygenase 1
TET2: Tet Methylcytosine Dioxygenase 2
TET3: Tet Methylcytosine Dioxygenase 3
TLBL: T-lymphoblastic lymphomas
TRIP13: Thyroid Hormone Receptor Interactor 13
TSS: Transcription start site
TTTY15: Testis Expressed Transcript, Y-Linked 15
TXK: TXK tyrosine kinase
UBAC2: UBA Domain Containing 2
UCK2: Uridine-cytidine kinase 2
UCON: Ultraconserved element
UCSC: University of California, Santa Cruz
UHRF1: Ubiquitin Like With PHD And Ring Finger Domains 1
USP9Y: Ubiquitin Specific Peptidase 9 Y-Linked
UTY: Ubiquitously Transcribed Tetratricopeptide Repeat Containing, Y-Linked
UXS1: UDP-Glucuronate Decarboxylase 1
VEGFA: Vascular Endothelial Growth Factor A
VEGFR2: Vascular endothelial growth factor receptor 2
WGBS: Whole Genome Bisulfite Sequencing
WHO: World Health Organization
ZWINT: ZW10 interacting kinetochore
